# A Genome-Wide Association Study of Total Bilirubin and Cholelithiasis Risk in Sickle Cell Anemia

**DOI:** 10.1371/journal.pone.0034741

**Published:** 2012-04-27

**Authors:** Jacqueline N. Milton, Paola Sebastiani, Nadia Solovieff, Stephen W. Hartley, Pallav Bhatnagar, Dan E. Arking, Daniel A. Dworkis, James F. Casella, Emily Barron-Casella, Christopher J. Bean, W. Craig Hooper, Michael R. DeBaun, Melanie E. Garrett, Karen Soldano, Marilyn J. Telen, Allison Ashley-Koch, Mark T. Gladwin, Clinton T. Baldwin, Martin H. Steinberg, Elizabeth S. Klings

**Affiliations:** 1 Department of Biostatistics, Boston University School of Public Health, Boston, Massachusetts, United States of America; 2 Department of Medicine, Boston University School of Medicine, Boston, Massachusetts, United States of America; 3 The Pulmonary Center, Boston University School of Medicine, Boston, Massachusetts, United States of America; 4 McKusick-Nathans Institute of Genetic Medicine, Johns Hopkins University School of Medicine, Baltimore, Maryland, United States of America; 5 Department of Pediatrics, Division of Pediatric Hematology, Johns Hopkins University School of Medicine, Baltimore, Maryland, United States of America; 6 Clinical and Molecular Hemostasis Laboratory Branch, Division of Blood Disorders, National Center on Birth Defects and Developmental Disabilities, Centers for Disease Control and Prevention, Atlanta, Georgia, United States of America; 7 Vanderbilt School of Medicine, Nashville, Tennessee, United States of America; 8 Department of Medicine, Duke University Medical Center, Durham, North Carolina, United States of America; 9 Division of Pulmonary, Allergy and Critical Care Medicine and the Vascular Medicine Institute, University of Pittsburgh, Pittsburgh, Pennsylvania, United States of America; Instituto de Higiene e Medicina Tropical, Portugal

## Abstract

Serum bilirubin levels have been associated with polymorphisms in the *UGT1A1* promoter in normal populations and in patients with hemolytic anemias, including sickle cell anemia. When hemolysis occurs circulating heme increases, leading to elevated bilirubin levels and an increased incidence of cholelithiasis. We performed the first genome-wide association study (GWAS) of bilirubin levels and cholelithiasis risk in a discovery cohort of 1,117 sickle cell anemia patients. We found 15 single nucleotide polymorphisms (SNPs) associated with total bilirubin levels at the genome-wide significance level (p value <5×10^−8^). SNPs in *UGT1A1, UGT1A3, UGT1A6, UGT1A8* and *UGT1A10*, different isoforms within the *UGT1A* locus, were identified (most significant rs887829, p = 9.08×10^−25^). All of these associations were validated in 4 independent sets of sickle cell anemia patients. We tested the association of the 15 SNPs with cholelithiasis in the discovery cohort and found a significant association (most significant p value 1.15×10^−4^). These results confirm that the *UGT1A* region is the major regulator of bilirubin metabolism in African Americans with sickle cell anemia, similar to what is observed in other ethnicities.

## Introduction

Cholelithiasis is the most common gastrointestinal disease to require hospitalization worldwide [1]. Most gallstones are primarily comprised of cholesterol; however, in certain patient risk groups, bile pigment stones are extremely common and represent a significant cause of morbidity. These stones are associated with increased serum unconjugated bilirubin levels, which occur in a number of conditions including hemolytic anemia. In other patient groups, ineffective erythropoiesis, ileal diseases, or gastrointestinal resections producing increased colonic spillage of bile salts, predispose to bile pigment stones [2].

Patients with sickle cell disease (SCD), particularly those with sickle cell anemia (homozygosity for *HBB* glu6val), are at risk for bile pigment cholelithiasis due to the association of this disease with hemolysis which produces an unconjugated hyperbilirubinemia [3]. Cholelithiasis occurs in up to 70% of these patients, resulting from the precipitation of polymerized calcium bilirubinate within the gallbladder lumen when the ion product of calcium and unconjugated bilirubin exceeds its solubility. Up to 50% of these patients undergo cholecystectomy at some point during their lifetimes, suggesting increased associated morbidity, creating a need for hospitalization, and potential mortality from infectious or other complications related to this process(1).

Cholelithiasis risk, for both cholesterol and bile pigment stones, is thought to be partially mediated by genes impacting bilirubin metabolism [4], [5], [6]. Genome-wide association studies (GWAS) of three large Caucasian cohorts have identified single nucleotide polymorphisms (SNPs) in *UGT1A1* that are responsible for 18% of clinical variability of serum bilirubin [7]. Sequencing of the *UGT1A1* gene in patients with Gilbert’s syndrome, associated with unconjugated hyperbilirubinemia, revealed that an important modulator of enzyme activity is the length of TA repeats in the promoter region; the greater the number of repeats being associated with the highest serum bilirubin levels and cholelithiasis risk [8], [9].

Genome-wide studies examining hyperbilirubinemia and cholelithiasis risk have not been performed in persons of African descent with sickle cell anemia. However, sequencing of *UGT1A1* has been undertaken in SCD cohorts in Guadeloupe, Portugal, Jamaica, India and the United States; genotyping of 324 SCD children from the Cooperative Study of Sickle Cell Disease (CSSCD) revealed that the common (TA)6 and (TA)7 alleles were present in 75% [10]–[15]. The (TA)7/(TA)7 genotype was associated with increased serum bilirubin levels and cholelithiasis risk compared with the (TA)6/(TA)7 and (TA)6/(TA)6 genotypes across cohorts with suggestions that those with a (TA)8 allele are at highest risk, suggesting a similar pattern of inheritance as observed in Caucasians [10]–[15]. Treatment with hydroxyurea, known to decrease the hemolytic rate in sickle cell anemia patients, was unable to decrease bilirubin levels to normal levels in those with the (TA)7/(TA)7 genotype suggesting that this genetic defect may represent a new pharmacologic target for these patients [14].

**Table 1:** Patient Characteristics in CSSCD, MSH and Walk-PHaSST cohorts.

Clinical Variable	CSSCD			MSH			Walk-PHaSST		
	Overall(N = 1117)	Men (N = 533)	Women (N = 584)	Overall (N = 195)	Men (N = 96)	Women (N = 99)	Overall (N = 522)	Men (N = 241)	Women (N = 281)
Log Total Bilirubin (mg/dL)*	1.13(0.5)	1.19(0.5)	1.13(0.5)	1.09(0.6)	1.15(0.6)	1.04(0.6)	3.67(0.7)	3.81(0.7)	3.55(0.7)
Reticulocyte (%)	12.09(5.9)	11.79(5.6)	12.09(5.9)	15.06(8.1)	14.13(7.8)	15.90(8.3)	8.61(5.5)	8.99(5.6)	8.28(5.5)
AST (units/dL)	50.52(32.2)	48.68(34.5)	50.52(32.2)	41.45(19.3)	42.03(19.1)	40.91(19.6)	46.27(34.9)	51.55(41.4)	41.77(27.4)
ALT (units/dL)	22.14(22.6)	22.25(24.7)	22.14(22.6)	24.14(15.8)	24.81(15.6)	23.52(16.1)	28.33(22.4)	30.23(23.3)	26.61(21.5)
Hemoglobin (g/dL)	8.43(8.5)	8.49(1.3)	8.43(8.5)	8.56(1.4)	9.01(1.4)	8.14(1.3)	9.37(2.0)	9.63(2.1)	9.14(1.8)
LDH (mg/dL)	421.1(91.1)	438.1(88.4)	421.1(91.1)	NA	NA	NA	454.43(292.6)	493.05(316.4)	421.92(267.2)
Age (years)	16.41(11.5)	15.29(11.1)	16.41(11.5)	28.61(6.2)	28.61(6.2)	30.28(8.4)	36.69(13.2)	34.87(13.1)	38.25(13.2)

* Walk-PHaSST bilirubin measurement is in SI units.

Summary statistics of patient characteristics in the CSSCD, MSH, and WALK-PHaSSTstudies. For each study, the first column reports statistics (mean and standard deviation) for all patients included in the analysis and the second and third columns report statistics stratified by gender.

**Table 2:** Patient Characteristics of the Duke and SITT cohorts.

Clinical Variable	Duke			SITT		
	Overall (N = 530)	Men (N = 242)	Women (N = 288)	Overall (N = 905)	Men (N = 480)	Women (N = 425)
Log Total Bilirubin (mg/dL)*	0.83 (0.7)	0.89 (0.8)	0.78 (0.7)	1.12(0.6)	1.16(0.6)	1.08(0.6)
Reticulocyte (%)	10.35 (6.0)	10.22 (6.0)	10.45 (6.1)	11.89(5.5)	12.01(5.6)	11.75(5.4)
AST (units/dL)	46.56 (34.6)	47.69 (29.2)	45.61 (38.5)	NA	NA	NA
ALT (units/dL)	30.93 (28.2)	32.41 (28.7)	29.69 (27.8)	NA	NA	NA
Hemoglobin (g/dL)	8.75 (1.8)	9.20 (2.1)	8.40 (1.6)	8.12(1.1)	8.01(1.1)	8.26(1.1)
LDH (mg/dL)	NA	NA	NA	NA	NA	NA
Age (years)	33.67 (11.9)	32.34 (11.0)	34.79 (12.6)	8.96(2.4)	9.07(2.5)	8.84(2.4)

Summary statistics of patient characteristics in the DUKE and SITT studies. For each study, the first column reports statistics (mean and standard deviation) for all patients included in the analysis and the second and third columns report statistics stratified by gender.

**Figure 1:**
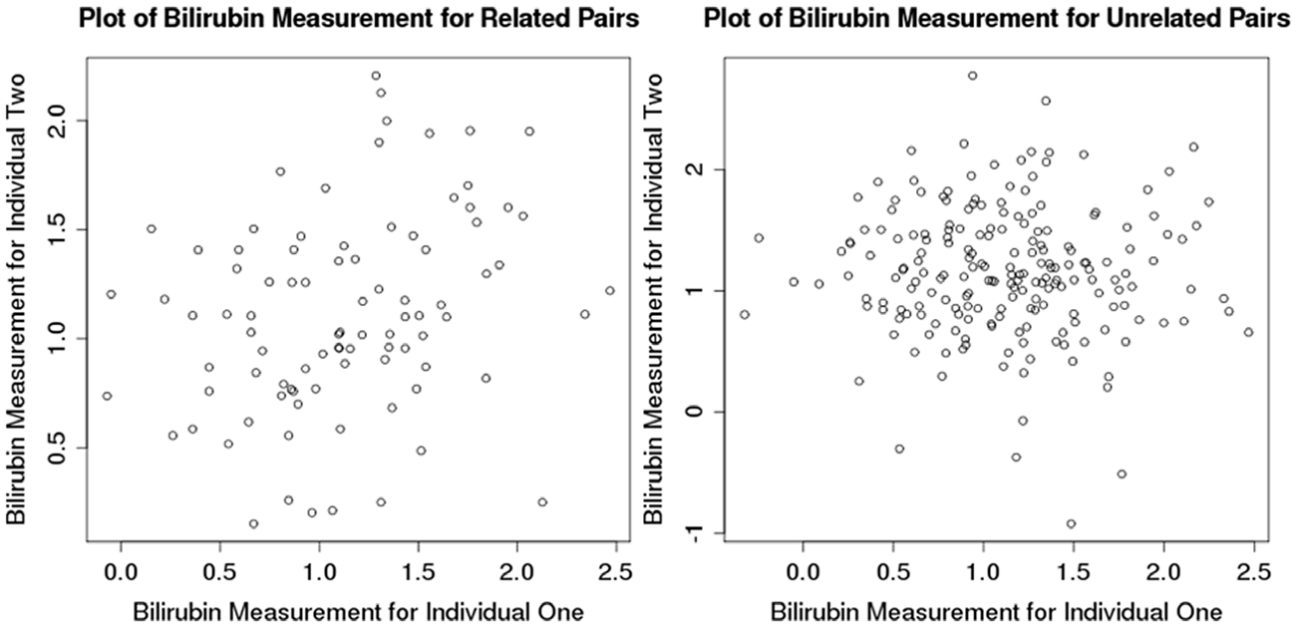
Plot of serum bilirubin among 90 sibling pairs in the CSSCD (A) and 200 pairs of unrelated individuals randomly selected from the CSSCD (B). In each scatter plot, the x- and y-axes show levels of total bilirubin. The correlation coefficient in the 90 sibling pairs was 0.27, while the average correlation coefficient of bilirubin levels in the pairs of unrelated individuals was −0.002.

One of the reasons for the focus on *UGT1A1* in genetic studies relates to its known importance in bilirubin metabolism [8], [9]. Upon release into the plasma, heme is metabolized to bilirubin via the actions of heme oxygenase-1. Bilirubin is poorly water soluble and in order for excretion to occur, disruption of its hydrogen bonds via a process termed glucuronidation, or conjugation, is essential. Glucuronidation of bilirubin is mediated by a family of enzymes termed the uridine-diphosphoglucuronate glucuronosyltransferases (UGT). Of these, *UGT1A1* is the most important regulator of this process clinically and is one of the isoforms of the *UGT1A* gene complex comprised of 9 transcriptional units. Unconjugated or indirect bilirubin is responsible for all of the toxic effects of hyperbilirubinemia, supporting the clinical importance of this class of enzymes. Additionally, as alluded to previously, genetic studies of other patient populations characterized by hyperbilirubinemia, Gilbert’s syndrome and cystic fibrosis, have demonstrated an association between genetic variants within dinucleotide repeats in the promoter of *UGT1A1* and bile pigment stone formation [7].

**Figure 2:**
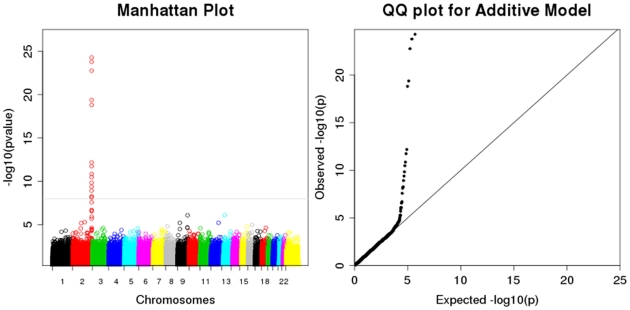
Summary of the GWAS data from the CSSCD Cohort. The Manhattan plot (A) displays the –log10(p value) of the associations tested in the CSSCD cohort using the additive model. Color bands represent chromosomes, and SNPs are ordered by their physical position within each chromosome. The large spike in chromosome 2 corresponds to the *UGT1A1, UGT1A3, UGT1A8* and *UGT1A10* regions. The QQ-plot (B) displays the observed (y-axis) versus expected (x-axis) –log10 (p-value). From the QQ plot, there is minimal to no inflation in the test statistic.

**Table 3:** Single Nucleotide Polymorphisms Associated with Total Bilirubin Levels.

Variant Information							CSSCD		MSH		WALK-PHaSST		Duke		SITT	
SNP	Chr	BP	Coded Allele	Noncoded Allele	MAF	Genes	Β	pvalue	β	pvalue	β	pvalue	β	pvalue	β	pvalue
rs7586110	2	234255266	C	A	0.2632	UGT1A6–UGT1A10*	0.11	2.63E−08	0.07	.03347	.20	.000107	0.21	2.20E−05	0.24	8.65E−14
rs10168155	2	234261575	A	G	0.3735	UGT1A6–UGT1A10*	0.11	5.72E−09	NA	NA	0.18	0.000164	0.26	4.44E−09	0.24	3.52E−16
rs10168416	2	234261826	G	C	0.2402	UGT1A6–UGT1A10*	0.12	5.55E−09	NA	NA	0.21	4.46E−05	0.21	4.03E−05	0.26	1.16E−13
rs6759892	2	234266408	C	A	0.3766	UGT1A6–UGT1A10*	0.10	7.71E−09	0.08	.0045	.18	.000112	0.26	4.44E−09	0.24	7.31E−17
rs1105880	2	234266704	G	A	0.3628	UGT1A6–UGT1A10*	0.11	1.20E−09	NA	NA	0.18	0.000121	0.27	1.84E−09	0.26	3.13E−18
rs2070959	2	234266930	G	A	0.2468	DNAJB3,UGT1A3–UGT1A10ψ	0.13	4.05E−10	0.07	.05408	0.21	5.56E−05	0.22	1.62E−05	0.26	1.24E−15
rs1105879	2	234266941	C	A	0.3024	UGT1A6–UGT1A10*	0.13	1.39E−11	0.07	.02737	0.18	.000189	0.24	1.10E−06	0.28	7.68E−20
rs17863787	2	234275833	C	A	0.2498	UGT1A6–UGT1A10*	0.14	3.34E−11	NA	NA	0.28	1.22E−08	0.22	8.75E−06	0.28	4.93E−18
rs3755319	2	234332321	A	C	0.2913	UGT1A1–UGT1A10 ψ	−0.14	1.82E−12	NA	NA	−0.26	5.59E−08	−0.29	5.07E−09	−0.28	2.21E−18
rs887829	2	234333309	A	G	0.4532	UGT1A1–UGT1A10 ψ	0.19	5.27E−25	NA	NA	0.37	1.56E−18	0.33	2.30E−14	0.41	3.16E−46
rs6742078	2	234337378	A	C	0.433	UGT1A1–UGT1A10 ψ	0.18	1.71E−23	NA	NA	0.36	3.67E−17	0.33	3.18E−14	0.38	1.43E−43
rs4148324	2	234337461	C	A	0.4459	UGT1A1–UGT1A10 ψ	0.16	1.56E−19	NA	NA	0.35	4.89E−16	0.33	4.58E−14	0.37	2.55E−38
rs3771341	2	234337978	A	G	0.3998	UGT1A1–UGT1A10 ψ	0.17	4.17E−20	NA	NA	0.35	1.53E−15	0.28	2.08E−10	0.39	1.26E−37
rs4148325	2	234338048	A	G	0.4525	UGT1A1–UGT1A10 ψ	0.18	1.66E−24	NA	NA	0.37	2.13E−18	0.33	2.36E−14	0.40	2.11E−47
rs4148326	2	234338201	A	G	0.3874	UGT1A1–UGT1A10 ψ	−0.13	6.57E−13	NA	NA	−0.28	7.17E−11	−0.27	3.80E−09	−0.31	5.44E−26

Genome wide significant SNPs in the CSSCD study and their replicates in the independent cohorts. The table reports the SNP identifier from dbSNP, chromosome, physical coordinates (hg18), the coded allele in PLINK (also minor allele) and the non-coded allele, the minor allele frequency (MAF) from the CSSCD cohort, the gene clusters where the SNP is located, and regression coefficientt and p-value in each study. Additive models of association were used in all studies. NA in the MSH means the SNP was unavailable in the 370 Illumina array.

**Figure 3:**
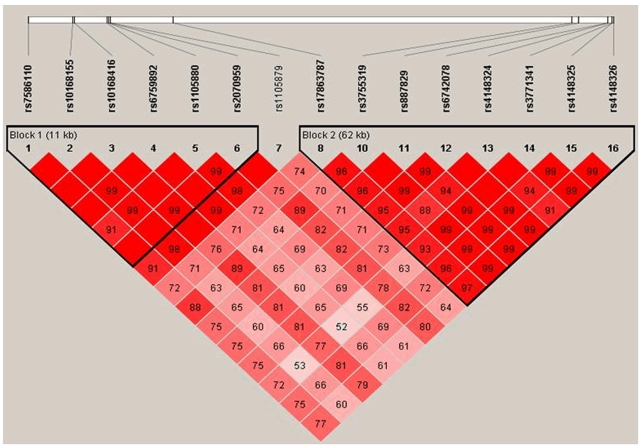
LD Structure in the CSSCD Cohort. LD plots for regions in genes *UGT1A1, UGT1A3, UGT1A4, UGT1A5, UGT1A6, UGT1A7, UGT1A8, UGT1A9* and *UGT1A10* on chromosome 2 in the CSSCD subjects. The LD plot was generated using Haploview 4.2. Each diamond represents the D’ value between two SNPs. The LD color scheme is: white D’<1 and LOD<2, blue D’ = 1 and LOD<2; shades of pinkish-red D’<1 and LOD≥2 and bright red D’ = 1 and LOD≥2.

We conducted a GWAS of serum total bilirubin in the largest number of African American sickle cell anemia patients studied to date to determine if genetic variants played a role in the increased risk of cholelithiasis observed in this population. Our analysis shows that *UGT1A1* is the major regulator of bilirubin levels in African Americans with sickle cell anemia, and that genetically mediated differences in bilirubin conjugation play an important role in cholelithiasis risk in these patients.

## Materials and Methods

### Ethics Statement

All studies were approved by the Institutional Review Board of Boston Medical Center, Duke University, University of Pittsburgh, Johns Hopkins University, Washington University and the University of North Carolina at Chapel Hill. Additionally, IRB approval was acquired from all of the sites participating in the Walk-PHaSST, Multicenter Study of Hydroxyurea Use and SITT trials for subject enrollment. All patients signed informed written consent for participation in these studies.

**Table 4:** Single Nucleotide Polymorphisms Associated with Cholelithiasis.

SNP	Chr	BP	Coded Allele	Non-Coded Allele	MAF	OR	P value
rs7586110	2	234255266	C	A	0.2632	1.366	0.05779
rs10168155	2	234261575	A	G	0.373	1.393	0.02858
rs10168416	2	234261826	G	C	0.2402	1.417	0.03765
rs6759892	2	234266408	C	A	0.3763	1.415	0.02134
rs1105880	2	234266704	G	A	0.3628	1.408	0.02277
rs2070959	2	234266930	G	A	0.2468	1.4	0.04248
rs1105879	2	234266941	C	A	0.303	1.356	0.05056
rs17863787	2	234275833	C	A	0.2498	1.324	0.09915
rs3755319	2	234332321	A	C	0.2913	0.561	0.001284
rs887829	2	234333309	A	G	0.4532	1.715	0.00032
rs6742078	2	234337378	A	C	0.433	1.635	0.001044
rs4148324	2	234337461	C	A	0.4459	1.654	0.000878
rs3771341	2	234337978	A	G	0.3998	1.661	0.00087
rs4148325	2	234338048	A	G	0.4525	1.757	0.000175
rs4148326	2	234338201	A	G	0.3874	0.524	0.000115

Results of SNP association analysis with cholelithiasis in the CSSCD study using the additive model. The minor allele is the coded allele, and the OR is the odds for cholelithiasis in carriers of one extra copy of the coded allele.

**Table 5:** Association Analysis with LDH, Reticulocyte Counts and Hemoglobin Concentration.

SNP	LDH		Reticulocytes		Hemoglobin	
	β	pval	β	pval	β	pval
rs7586110	−0.01206	0.235	0.01255	0.4964	0.02221	0.6891
rs10168155	−0.00587	0.5304	−0.0032	0.8501	0.04552	0.3687
rs10168416	−0.0042	0.6886	0.01099	0.563	0.02353	0.6808
rs6759892	−0.00604	0.5171	0.001128	0.9466	0.04249	0.4034
rs1105880	0.000316	0.9729	7.16E−05	0.9966	0.04007	0.4277
rs2070959	−0.00319	0.7579	0.01636	0.3834	0.003175	0.9552
rs1105879	−0.00135	0.8891	0.02488	0.1554	0.0182	0.7298
rs17863787	0.00116	0.9146	0.006013	0.7586	0.004455	0.9399
rs3755319	−0.00043	0.9653	0.006524	0.7188	0.01732	0.7509
rs887829	−0.00528	0.558	−0.01726	0.2922	0.06559	0.1825
rs6742078	−0.00786	0.3855	−0.01868	0.2558	0.05936	0.2303
rs4148324	−0.00463	0.6104	−0.02468	0.1339	0.06086	0.2193
rs3771341	−0.00911	0.3252	−0.01876	0.2654	0.07966	0.1158
rs4148325	−0.00799	0.3732	−0.02165	0.1877	0.06967	0.1576
rs4148326	0.003204	0.7309	0.01245	0.462	0.02886	0.5721

Results of SNP association analysis with other hemolytic phenotypes including hemoglobin, LDH and reticulocyte count in the CSSCD Study.

### Study Subjects

The discovery set consisted of 1,117 African American subjects from the CSSCD [16], [17]. The replication cohorts were comprised of 195 subjects from the Multicenter Study of Hydroxyurea (MSH), 522 subjects from the Pulmonary Hypertension and Sickle Cell Disease with Sildenafil Therapy (Walk-PHaSST) study (NCT00492531) [18], 530 subjects from the Outcome Modifying Genes study (referred to subsequently as “Duke”) that enrolled patients from Duke University Medical Center, the University of North Carolina Chapel Hill, Emory University, East Carolina University Comprehensive Sickle Cell Centers, and the Carolinas Health Center, and 905 samples from the SITT silent cerebral infarct trial (NCT00072761). The timing of each of these studies impacted hydroxyurea use. None of the patients from the CSSCD discovery set were treated with hydroxyurea as recruitment for this study concluded in 1998. To eliminate potential confounding effects of hydroxyurea, in the MSH, pre-hydroxyurea levels of total bilirubin were utilized [19].

### Genotyping

The DNA from the CSSCD, MSH, and Walk-PHaSST samples were genotyped at Boston University using the Illumina Human610-Quad SNP array, with approximately 600,000 SNPs and the Illumina HumanCNV370-duo bead chip (MSH). The Duke samples were also genotyped using the Human610-Quad SNP array, whereas the SITT cohort was genotyped in two stages. In stage 1, a subset of 573 recruited samples was genotyped at the Center for Inherited Disease Research (CIDR) using IIIumina HumanHap650Y array. The genotype data for the remaining 509 samples were generated at the Center for Disease Control (CDC) in collaboration with Washington University using Illumina Infinium HumanOmni1-Quad array. To infer un-typed SNPs and fill-in missing data across Omni1M and HumanHap650Y platforms, imputation was performed by using Hidden Markov model implemented in MaCH software [20]. All samples genotyped at Boston University were processed according the manufacturer’s protocol and BeadStudio Software was used to make genotype calls utilizing the Illumina pre-defined clusters. Samples with less than a 95% call rate were removed and SNPs with a call rate <97.5% were re-clustered. After re-clustering, SNPs with call rates >97.5%, cluster separation score >.25, excess heterozygosity between −.10 and .10, and minor allele frequency >5% were retained in the analysis. We used the genome-wide identity by descent analysis in PLINK to discover unknown relatedness. Pairs with identity by descent measurements greater than .2 were deemed to be related subjects. Related subjects within individual or different studies were removed. We also removed samples with inconsistent gender findings defined by heterozygosity of the X chromosome and gender recorded in the database. Quality control parameters for the DUKE and SITT samples were comparable. In SITT, a 98% cutoff was used for both the sample and SNP call rates in the combined dataset (n = 1082), and resulted the exclusion of 6 individuals and 1,260 SNPs from the study. Seventy seven samples were identified as first-degree relatives (pairs with identity by descent measurements > 0.4), and 1 individual from each pair was removed. Twenty six individuals were identified as genetic outliers using EIGENSTRAT (>6 standard deviations on any of the top ten principal components) and removed. Additionally, 68 samples were also dropped from the study, due to missing phenotype data, leaving 905 samples for subsequent analysis.

### Phenotype

Serum total and direct bilirubin, lactate dehydrogenase (LDH), hemoglobin concentrations, and reticulocyte counts were measured using automated chemical and hematologic analyzers at the individual medical centers participating in these studies.

For the CSSCD patients, longitudinal bilirubin measurements were collected from phases 1, 2, and 3 of the study [16]. Only steady state measurements were used (4 months removed from blood transfusion). The longitudinal measurements of 3,250 study patients were analyzed using a Bayesian hierarchical mixed model that included a random effect per patient to account for the repeated measurements, as well as random intercept and age effects that were allowed to vary with the clinics. The random intercept and age effects were used to remove the between-site systematic differences. Markov Chain Monte Carlo method in Openbugs was used to estimate the predicted total bilirubin values, and log-transformed median predicted values were used as the phenotype for the GWAS. The details of the analysis are available as (Information S1). For MSH, Duke, Walk-PHaSST and SITT patients, the log transformed baseline total bilirubin was used.

CSSCD subjects were assessed for gallstones by cholecystogram, plain film report, and abdominal ultrasound. A combination of questionnaires and medical records were utilized to determine a history of cholelithiasis or cholecystectomy. The cholelithiasis phenotype was dichotomized into: a) those who had gallstones (or history of), a history of cholecystectomy, a nonfunctional gallbladder; or b) none of these conditions. Of the 1,062 CSSCD patients with gallstone information, 348 had gallstones, or a nonfunctional gallbladder, cholecystectomy or a history of one of these events.

### Heritability of Total Bilirubin

To further examine heritability of serum bilirubin in the CSSCD population, we examined the correlation of serum bilirubin in 90 sibling pairs. As a comparison, we randomly selected serum bilirubin values for 200 unrelated pairs 1,000 times to get an average of the estimated correlation among unrelated individuals.

### Description of GWAS and Secondary Genetic Association Analyses

The association between total bilirubin levels and each SNP that passed quality control was tested using multiple linear regression, adjusting for age and gender using the additive and genotypic model in PLINK software. Age and gender were both included as covariates, as both were found to have a significant association with total bilirubin levels (p<0.0001 for both covariates). The minor allele was the coded allele in the additive model. To control for population stratification, a separate GWAS was performed on the discovery set adjusting for age, gender and the top ten principal components calculated using EIGENSOFT [21].

Haploview was employed to generate LD heatmaps using genotype data from the CSSCD. The SNPs that reached genome-wide significance in the CSSCD sample were analyzed for association in the other cohorts. In SITT, the genetic associations were performed after adjustments for the same covariates (age, gender and top ten principal components). To account for the uncertainty of imputed data, the estimated allele dosage was analyzed using ProABEL under a linear regression framework [22]. Duke only controlled for age and gender, because previous analyses had indicated there was not appreciable population stratification in that data set. In a secondary analysis, SNPs that achieved genome-wide significance from the additive model were tested for association with the cholelithiasis in the CSSCD cohort. This association was tested using an additive multiple logistic regression model, adjusting for age. The same SNPs were also tested for association with LDH, reticulocyte counts and hemoglobin concentration, using the same Bayesian hierarchical model.

## Results

### Patient Characteristics and Heritability of Total Bilirubin Levels

Patient characteristics for each cohort are shown in Tables 1 and 2. The CSSCD cohort was younger than the MSH, Duke, and Walk-PHaSST cohorts. Each of the cohorts were equally divided between males and females. Examination of the serum bilirubin levels amongst 90 sibling pairs revealed a positive correlation (r = 0.270, p = 0.009), consistent with heritability of bilirubin levels (Figure 1A). In contrast, there was no correlation amongst 1,000 randomly selected pairs (r = −.002 average p value = 0.502, Figure 1B).

### Association Between *UGT* SNPs and Serum Bilirubin Levels

After our quality control procedures, 569,615 SNPs were analyzed in the GWAS of serum bilirubin in the CSSCD. Figure 2A shows the Manhattan plot, with the results of the GWAS for the additive model and the large spike of significant associations in chromosome 2. The QQ plot (Figure 2B) shows no inflation (lambda factor, λ = 1.01) suggesting that there is no confounding by population stratification. To confirm this, we repeated the analysis after adjustment for the top ten principal components and the magnitude and significance of the top SNPs did not change. Fifteen SNPs reached genome-wide significance for both the genotypic and additive models and we report only the results from the additive model (Table 3). These genes are located on a contiguous part of chromosome 2 of the *UGT1* region. The linkage disequilibrium (LD) structure of these SNPs in Figure 3 shows two blocks in LD, although all 15 SNPs are in very strong LD (D’>0.53) (the LD structure of these SNPs can be show in terms of r^2^ in Information S1). To determine if these 15 SNPs were part of one or multiple independent signals, another GWAS was performed adjusting for age, sex and our top SNP, rs887829. None of the fourteen SNPs showed a significant association with serum bilirubin after this adjusted analysis (complete results can be seen in Information S1), thus suggesting that these 15 SNPs are a part of the same signal. A complete list of the SNPs with a p value less than 5×10^−05^ consistent with genome-wide significance can be found in Information S1. An increase in the number of minor alleles in all SNPs except rs4148326 and rs3755319 was associated with an increase in log total bilirubin (effect sizes ranging from 0.108 to 0.1838), so that the less common alleles are the risk alleles; however, the other two SNPs showed effect estimates in the opposite direction (effect sizes ranging from −.13 to −.14), so that the minor alleles are associated with lower levels of serum bilirubin. Only four of the SNPs that reached genome-wide significance in the CSSCD population were available for genotyping on the 370K array used for the MSH samples, while all of them were genotyped in the Duke, Walk-PHaSST and SITT cohorts. All of the SNPs were significantly associated with bilirubin levels and had a regression coefficient in the same direction in both the discovery and replication sets (Table 3).

### SNPs Associated with Bilirubin Levels are also Associated with Cholelithiasis

Table 4 shows the association between the 15 genome-wide significant SNPs and the odds for cholelithiasis. Twelve of the 15 SNPs were significantly associated with cholelithiasis (p <.05) and all of them had associated odds ratios for cholelithiasis in a direction consistent with that observed in the analysis of total bilirubin: An increase in the number of risk alleles for all SNPs except rs4148326 and rs3755319 was associated with an increased odds for cholelithiasis (odds ratios ranging from 1.324 to 1.757). The other two SNPs showed effect estimates in the opposite direction (odds ratios of .524 and .561). We also tested whether these 15 SNPs were significantly associated with LDH levels, hemoglobin concentration and reticulocyte count to see if there was an association with markers of hemolysis. None of the SNPs showed an association with LDH, hemoglobin concentration or reticulocyte count (Table 5).

## Discussion

Intravascular hemolysis in sickle cell anemia patients produces increased circulating heme levels and has been associated with clinical complications including cholestatic jaundice and cholelithiasis [10], [23]–[25], [26]–[28] Unconjugated hyperbilirubinemia is a risk factor for the development of bile pigment stones in sickle cell disease and other hemolytic anemias [12], [29]–[41]. Typically, bilirubin is formed from metabolism of heme, which is derived principally from erythrocytic hemoglobin. Heme is oxidatively metabolized by heme oxygenase-1 into biliverdin, which is subsequently reduced enzymatically by biliverdin reductase to bilirubin. Upon formation, bilirubin is water insoluble, due to the presence of internal hydrogen bonding that creates a contorted molecule structure, inhibiting solubility. This form of bilirubin, termed unconjugated or indirect bilirubin, is responsible for all of the toxic effects of bilirubin observed clinically. To permit excretion, bilirubin complexes with albumin and is transported via the bloodstream to the liver, where it undergoes glucuronidation by the UGT family of enzymes; *UGT1A1* is the only one with clinical relevance in humans [42]. *UGT1A* is a gene complex composed of 9 transcriptional units encoding an isoform of the *UGT* gene [30], [31]. The most common genetic cause of impaired bilirubin glucuronidation occurs in Gilbert’s syndrome, which follows an autosomal recessive inheritance pattern and most commonly affects Caucasians of European descent [43], [44]. Clinically, these patients have a mild indirect hyperbilirubinemia, which worsens during periods of stress or febrile illnesses [45]. Genetic studies of these patients have identified a dinucleotide repeat polymorphism (TA)_5–8_ in the TATA box of the *UGT1A1* gene promoter that is associated with reduced *UGT* expression and produces hyperbilirubinemia [30]–[31], [37], [46]–[47]. This polymorphism has also been observed in small cohorts of sickle cell anemia patients suggesting a common pathogenic link between ethnically divergent etiologies of indirect hyperbilirubinemia [5], [10]–[15], [33]. In the current study, genome-wide genetic screening of a large cohort of sickle cell anemia patients not only confirmed these findings but also supported their biological relevance.

In addition to defects in bilirubin metabolism, genetic alterations in the glucuronidation pathway have been linked to abnormalities in the hepatic metabolism of certain medications, pre-disposition to cardiovascular disease and certain types of malignancy suggesting a clinical level of importance beyond cholelithiasis [35], [42], [48]–[53]. How this relates to the pathogenesis of sickle cell anemia is not entirely clear clinically, but may be worthy of future study.

In genetic studies of primarily Caucasian cohorts, cholelithiasis and serum bilirubin levels are heritable traits. Family-based studies show that the *UGT* locus accounted for a significant proportion of the variation observed in both of these variables [18], [54]–[56]. Our study is the first to use GWAS to examine an African American population with sickle cell anemia, a condition where hemolysis predisposes patients to hyperbilirubinemia, to determine the heritability of serum bilirubin levels. Fifteen SNPs in *UGT1A1, UGT1A3, UGT1A4, UGT1A5, UGT1A6, UGT1A7, UGT1A8* and *UGT1A9* reached genome-wide significance for association with total bilirubin levels in the CSSCD cohort; 13 of these were also associated with the presence or history of cholelithiasis. These findings were confirmed in 4 other cohorts totaling 3,269 patients, unusual for a rare disease such as sickle cell anemia, and representing a greater number of patients than all of the previous studies of this population combined.

SNPs we identified in the current study have been linked to hyperbilirubinemia in other populations. SNP rs887829, the most significant SNP associated with bilirubin levels identified in the CSSCD, Duke and Walk-PHaSST cohorts, was previously observed in associated with total bilirubin levels in a GWAS of 4,300 Sardinians [57]. This study also reported a significant association with bilirubin and the *SLCO1B3* locus; however, we were unable to replicate these results in our study due to unavailability or low MAF of the markers in our array. In a study performed by Cheng et al. examining genetic variants associated with serum bilirubin in 619 healthy African Americans revealed that the top SNP was rs887829 (p = 1.77×10^−22^) [58]. This suggests that this variant is not unique to African Americans with sickle cell disease. It has been theorized that this SNP may confer protection against malaria which may explain its penetrance amongst African populations. SNP rs887829 is located in the promoter region of *UGT1A1*, 221 bp upstream from the (TA)_n_ repeat sequence. Horsfall et al. found a strong association was found between rs887829 and the (TA)_7_ and (TA)_8_ repeat sequence suggesting that this may be a marker for the (TA)7/(TA)7 or 8 genotypes [34]. This proximity to the promoter element and strong level of association suggest that rs887829 is a marker for the (TA)_n_ repeats and that additional sequencing of this gene would be redundant [58]. SNP rs6742078, identified in the current study, was associated with total bilirubin in a meta-analysis previously performed by Johnson et al., including the Framingham Heart Study, Rotterdam Study and Age, Gene, Environment and Susceptibility-Reykjavik study cohorts [33]. In addition, SNPs rs3755319, rs7586110, and rs6759892 were also found to be significantly associated with total bilirubin in a cohort of 4,300 Sardinians [57]. SNP rs887829 was found to be associated with cholelithiasis in a study published by Buch et al., in which a cohort of 2,606 German cholelithiasis patients was compared with 1,121 South American controls (OR = 1.73, p value .003) [11].

There was no association between LDH, hemoglobin concentration or reticulocyte count, markers of hemolysis, and the SNPs identified in the present study. A potential explanation for this is the weak association between bilirubin levels and hemolytic rate. While the "gold standard" for hemolysis is the red cell lifespan, this is rarely performed and surrogate blood biomarkers such as reticulocyte count, LDH, bilirubin and haptoglobin levels are used clinically to estimate the degree of hemolysis. None of these measures are specific for hemolysis. An elevated serum bilirubin level in sickle cell anemia reflects multiple pathophysiologic processes that include liver disease in addition to hemolysis and therefore may lack the phenotypic specificity required for genetic association studies of hemolysis. Another possible reason for this finding is that the genes associated with bilirubin levels are all involved in bilirubin catabolism and not production suggesting that they are not reflective of the processes leading to bilirubin formation.

In summary, SNPs in *UGT1A1* are most tightly associated with bilirubin levels in African Americans with sickle cell anemia. This study in conjunction with those conducted in other populations at risk for unconjugated hyperbilirubinemia and other cohorts of sickle cell anemia patients support the concept that genetically mediated differences in bilirubin conjugation play an important role in the cholelithiasis risk. It is possible that targeting this pathway pharmacologically may offer new therapeutic options for these patients.

## Supporting Information

Information S1 **Description of the Bayesian hierarchical model used to create the phenotype in the CSSCD cohort.** Supplementary Figure 1 contains information on the LD structure of the UGT1A region in the CSCCD cohort. Supplementary Table 1 contains information on the analysis of the association between bilirubin and after adjusting for our most significant SNP. Figure 1: LD Structure in CSSCD Cohort. LD plots for regions in genes *UGT1A1, UGT1A3, UGT1A4, UGT1A5, UGT1A6, UGT1A7, UGT1A8, UGT1A9* and *UGT1A10* on chromosome 2 in the CSSCD subjects. The LD plot was generated using Haploview 4.2. Each diamond represents the r^2^ value between two SNPs. The LD color scheme is: white r^2^ = 0, 0<r^2^<1 grey (the darker the shade of grey, the higher the r^2^ value), black r^2^ = 1.
(DOCX)

## References

[1] Adam S, Jonassaint J, Kruger H, Kail M, Orringer EP (2008). Surgical and obstetric outcomes in adults with sickle cell disease.. Am J Med.

[2] Au WY, Cheung WC, Chan GC, Ha SY, Khong PL (2003). Risk factors for hyperbilirubinemia and gallstones in Chinese patients with beta thalassemia syndrome.. Haematologica.

[3] Au WY, Cheung WC, Hu WH, Chan GC, Ha SY (2005). Hyperbilirubinemia and cholelithiasis in Chinese patients with hemoglobin H disease.. Ann. Hematol.

[4] Strassburg CP, Manns MP, Tukey RH (1998). Expression of the UDP-glucuronosyltransferase 1A locus in human colon. Identification and characterization of the novel extrahepatic UGT1A8.. J. Biol. Chem.

[5] Vasavda N, Menzel S, Kondaveeti S, Maytham E, Awogbade M (2007). The linear effects of alpha-thalassaemia, the UGT1A1 and HMOX1 polymorphisms on cholelithiasis in sickle cell disease.. Br. J. Haematol..

[6] Vogel A, Kneip S, Barut A, Ehmer U, Tukey RH (2001). Genetic link of hepatocellular carcinoma with polymorphisms of the UDP-glucuronosyltransferase UGT1A7 gene.. Gastroenterology.

[7] Benjamin EJ, Dupuis J, Larson MG, Lunetta KL, Booth SL (2007). Genome-wide association with select biomarker traits in the Framingham heart study.. BMC Med. Genet.

[8] Bosma PJ, Chowdhury JR, Bakker C, Gantla S, de Boer A (1995). The genetic basis of the reduced expression of bilirubin UDP-glucuronosyltransferase 1 in Gilbert’s syndrome.. N. Engl. J. Med..

[9] Bosma PJ, van der Meer IM, Bakker CT, Hofman A, Paul-Abrahamse M (2003). UGT1A1*28 allele and coronary heart disease: The Rotterdam study.. Clin. Chem..

[10] Chaar V, Keclard L, Diara JP, Leturdu C, Elion J (2005). Association of UGT1A1 polymorphism with prevalence and age at onset of cholelithiasis in sickle cell anemia.. Haematologica.

[11] Passon RG, Howard TA, Zimmerman SA, Schultz WH, Ware RE (2001). Influence of bilirubin uridine diphosphate-glucuronosyltransferase 1A promoter polymorphisms on serum bilirubin levels and cholelithiasis in children with sickle cell anemia.. J. Pediatr. Hematol. Oncol..

[12] Haverfield EV, McKenzie CA, Forrester T, Bouzekri N, Harding R (2005). UGT1A1 variation and gallstone formation in sickle cell disease.. Blood.

[13] Carpenter SL, Lieff S, Howard TA, Eggleston B, Ware RE (2008). UGT1A1 promoter polymorphisms and the development of hyperbilirubenemia and gallbladder disease in children with sickle cell anemia.. Am J Hematol.

[14] Italia KY, Jijina FF, Jain D, Merchant R, Nadkarni AH (2010). The effect of UGT1A1 promoter polymorphism on bilirubin response to hydroxyurea therapy in hemaglobinopathies.. Clin Biochem,.

[15] Martins R, Morais A, Dias A, Soares I, Rolao C (2008). Early modification of sickle cell disease clinical course by UDP-glucuronosyltransferase 1A1 gene promoter polymorphism.. J Hum Genet,.

[16] Went MS, Wethers D, Smith J, Steinberg MH (1992). Laboratory profile of sickle cell disease: a cross-sectional analysis. The Cooperative Study of Sickle Cell Disease.. Journal of Clinical Epidemiology.

[17] Sebastiani P, Solovieff N, Hartley SW, Milton JN, Riva A (2010). Genetic modifiers of the severity of sickle cell anemia identified through a genome-wide association study.. Am. J. Hematol..

[18] Machado RF, Barst RJ, Yovetich NA, Hassell KL, Kato GJ (2011). Hospitalization for pain in patients with sickle cell disease with sildenafil for elevated TRV and low exercise capacity.. Blood.

[19] Steinberg MH, Barton F, Castro O, Pegelow CH, Ballas SK (2003). Effect of hydroxyurea on mortality and morbidity in adult sickle cell anemia: Risks and benefits up to 9 years of treatment.. JAMA.

[20] Li Y, Willer CJ, Ding J, Scheet P, Abecasis GR (2010). MaCH: Using sequence and genotype data to estimate haplotypes and unobserved genotypes.. Genetic Epidemiology.

[21] Price AL, Patterson NJ, Plenge RM, Weinblatt ME, Shadick NA (2006). Principal components analysis corrects for stratification in genome-wide association studies.. Nature Genetics.

[22] Aulchenko YS, Struchalin MV, van Duijn CM (2010). ProbABEL package for genome-wide association analysis of imputed data.. BMC Bioinformatics.

[23] Buch S, Schafmayer C, Volzke H, Seeger M, Miquel JF (2010). Loci from a genome-wide analysis of bilirubin levels are associated with gallstone risk and composition.. Gastroenterology.

[24] Cecchin E, Innocenti F, D’Andrea M, Corona G, De Mattia E (2009). Predictive role of the UGT1A1, UGT1A7, and UGT1A9 genetic variants and their haplotypes on the outcome of metastatic colorectal cancer patients treated with fluorouracil, leucovorin, and irinotecan.. J. Clin. Oncol..

[25] Chaar V, Keclard L, Etienne-Julan M, Diara JP, Elion J (2006). UGT1A1 polymorphism outweighs the modest effect of deletional (–3.7 kb) alpha-thalassemia on cholelithogenesis in sickle cell anemia.. Am. J. Hematol..

[26] Reiter CD, Wang X, Tanus-Santos JE, Hogg N, Cannon RO (2002). Cell-free hemoglobin limits nitric oxide bioavailability in sickle-cell disease.. NatMed.

[27] Taylor JG 6th, Nolan VG, Mendelsohn L, Kato GJ, Gladwin MT (2008). Chronic hyper-hemolysis in sickle cell anemia: Association of vascular complications and mortality with less frequent vasoocclusive pain.. PLoS One.

[28] Van Erpecum KJ (2011). Pathogenesis of cholesterol and pigment gallstones: An update.. Clin. Res. Hepatol. Gastroenterol..

[29] Clementi M, Di Gianatonio E, Fabris L, Forbosco P, Strazzabosco M (2007). Inheritance of hyperbilirubinemia: Evidence for a major autosomal recessive gene.. Dig Liver Dis.

[30] Galanello R, Piras S, Barella S, Leoni GB, Cipollina MD (2001). Cholelithiasis and Gilbert’s syndrome in homozygous beta-thalassaemia.. Br. J. Haematol..

[31] Gaston M RW (1982). The cooperative study of sickle cell disease: Review of study design and objectives.. Am J Pediatr Hematol Oncol.

[32] Haider MZ, Ashebu S, Aduh P, Adekile AD (1998). Influence of alpha-thalassemia on cholelithiasis in SS patients with elevated Hb F. Acta Haematol..

[33] Heeney MM, Howard TA, Zimmerman SA, Ware RE (2003). UGT1A promoter polymorphisms influence bilirubin response to hydroxyurea therapy in sickle cell anemia.. J. Lab. Clin. Med..

[34] Horsfall LJ, Zeitlyn D, Tarekegn A, Bekele E, Thomas MG (2011). Prevalence of clinically relevant UGT1A alleles and haplotypes in African populations.. Annals of Human Genetics.

[35] Hunt SC, Kronenberg F, Eckfeldt JH, Hopkins PN, Myers RH (2001). Association of plasma bilirubin with coronary heart disease and segregation of bilirubin as a major gene trait: The NHLBI family heart study.. Atherosclerosis.

[36] Johnson AD, Kavousi M, Smith AV, Chen MH, Dehghan A (2009). Genome-wide association meta-analysis for total serum bilirubin levels.. Hum. Mol. Genet..

[37] Kama NA, Atli M, Doganay M, Kologlu M, Reis E (2001). Practical recommendations for the prediction and management of common bile duct stones in patients with gallstones.. Surg. Endosc..

[38] Kang TW, Kim HJ, Ju H, Kim JH, Jeon YJ (2010). Genome-wide association of serum bilirubin levels in Korean population.. Hum. Mol. Genet..

[39] Kato GJ, Gladwin MT, Steinberg MH (2007). Deconstructing sickle cell disease: Reappraisal of the role of hemolysis in the development of clinical subphenotypes.. Blood Rev..

[40] Kato GJ, McGowan V, Machado RF, Little JA, Taylor J (2006). Lactate dehydrogenase as a biomarker of hemolysis-associated nitric oxide resistance, priapism, leg ulceration, pulmonary hypertension, and death in patients with sickle cell disease.. Blood.

[41] Kohle C, Mohrle B, Munzel PA, Schwab M, Wernet D (2003). Frequent co-occurrence of the TATA box mutation associated with Gilbert’s syndrome (UGT1A1*28) with other polymorphisms of the UDP-glucuronosyltransferase-1 locus (UGT1A6*2 and UGT1A7*3) in Caucasians and Egyptians.. Biochem. Pharmacol..

[42] Perera MA, Innocenti F, Ratain MJ (2008). Pharmacogenetic testing for uridine diphosphate glucuronosyltransferase 1A1 polymorphisms: Are we there yet?. Pharmacotherapy.

[43] Persico M, Persico E, Bakker CT, Rigato I, Amoroso A (2001). Hepatic uptake of organic anions affects the plasma bilirubin level in subjects with Gilbert’s syndrome mutations in UGT1A1.. Hepatology.

[44] Teng HC, Huang MJ, Tang KS, Yang SS (2007). Combined UGT1A1 and UGT1A7 variant alleles are associated with increased risk of Gilbert’s syndrome in Taiwanese adults.. Clin. Genet..

[45] Krawczyk M, Wang DQ, Portincasa P, Lammert F (2011). Dissecting the genetic heterogeneity of gallbladder stone formation.. Semin Liver Dis.

[46] Kronenberg F, Coon H, Gutin A, Abkevich V, Samuels ME (2002). A genome scan for loci influencing anti-atherogenic serum bilirubin levels.. Eur J Hum Genet.

[47] Rantner B, Kollerits B, Anderwald-Stadler M, Klein-Weigel P, Gruber I (2008). Association between the UGT1A1 TA-repeat polymorphism and bilirubin concentration in patients with intermittent claudication: Results from the CAVASIC study.. Clin. Chem..

[48] Lammert F, Matern S (2005). The genetic background of cholesterol gallstone formation: An inventory of human lithogenic genes.. Curr. Drug Targets. Immune. Endocr. Metabol. Disord..

[49] Lammert F, Sauerbruch T (2005). Mechanisms of disease: The genetic epidemiology of gallbladder stones.. Nat. Clin. Pract. Gastroenterol. Hepatol..

[50] Lin JP, O’Donnell CJ, Schwaiger JP, Cupples LA, Lingenhel A (2006). Association between the UGT1A1*28 allele, bilirubin levels, and coronary heart disease in the Framingham heart study.. Circulation.

[51] Lingenhel A, Kollerits B, Schwaiger JP, Hunt SC, Gress R (2008). Serum bilirubin levels, UGT1A1 polymorphisms and risk for coronary artery disease.. Exp. Gerontol..

[52] Portincasa P, Moschetta A, Palasciano G (2006). Cholesterol gallstone disease.. Lancet.

[53] Schwertner HA, Vitek L (2008). Gilbert syndrome, UGT1A1*28 allele, and cardiovascular disease risk: Possible protective effects and therapeutic applications of bilirubin.. Atherosclerosis.

[54] Liu J, Yang XM, Liu G, Chang LS, Zhang LR (2009). Association between genetic polymorphism of UGT1A7 and susceptibility of bladder cancer.. Zhonghua Yi Xue Za Zhi.

[55] McDonagh AF (2010). Controversies in bilirubin biochemistry and their clinical relevance.. Semin Fetal Neonatal Med.

[56] Ockenga J, Vogel A, Teich N, Keim V, Manns MP (2003). UDP glucuronosyltransferase (UGT1A7) gene polymorphisms increase the risk of chronic pancreatitis and pancreatic cancer.. Gastroenterology.

[57] Sanna S, Busonero F, Maschio A, McArdle PF, Usala G (2009). Common variants in the SLCO1B3 locus are associated with bilirubin levels and unconjugated hyperbilirubinemia.. Hum. Mol. Genet..

[58] Chen G, Ramos E, Adeyemo A, Shriner D, Zhou J (2011). GWAS for serum bilirubin in African Americans.. European Journal of Human Genetics.

