# Supplementary material for: A Genome-Wide Association Study of Total Bilirubin and Cholelithiasis Risk in Sickle Cell Anemia
Source: PLoS One. 2012 Apr 27;7(4):e34741. doi: 10.1371/journal.pone.0034741 (PMC3338756; doi:10.1371/journal.pone.0034741)
Supplement: Information S1 — Description of the Bayesian hierarchical model used to create the phenotype in the CSSCD cohort. Supplementary Figure 1 contains information on the LD structure of the UGT1A region in the CSCCD cohort. Supplementary Table 1 contains information on the analysis of the association between bilirubin and after adjusting for our most significant SNP. Figure 1: LD Structure in CSSCD Cohort. LD plots for regions in genes UGT1A1, UGT1A3, UGT1A4, UGT1A5, UGT1A6, UGT1A7, UGT1A8, UGT1A9 and UGT1A10 on chromosome 2 in the CSSCD subjects. The LD plot was generated using Haploview 4.2. Each diamond represents the r2 value between two SNPs. The LD color scheme is: white r2 = 0, 0<r2<1 grey (the darker the shade of grey, the higher the r2 value), black r2 = 1. (DOCX) [file pone.0034741.s001.docx]

**Methods**

The phenotype used in the discovery set was longitudinal total serum bilirubin adjusted for clinic, sex and age using a Bayesian hierarchical model. These measurements are modeled using a lognormal distribution with mean, μ and variance, τ. The mean, μ was adjusted using the following formula:

μ = β_0,clinic_+β_clinic,age_(age-mean(age)) + β_clinic,sex_(sex-mean(sex)) + β_patient_

The mean of distribution was modeled such that each clinic has a separate slope and age and gender effect. The patient ,age and gender random effect and intercept were modeled using a normal prior.

β_patient_~dnorm(0,τ_patient_)

β_0,clinic_~dnorm(alpha.0,τ_0_)

β_age_~dnorm(alpha.age,τ_age_)

β_sex_~dnorm(alpha.sex,τ_sex_)

The mean of the random effects was modeled with a normal distribution and the variance a gamma prior:

alpha.0~ dnorm(200,0.0001)

alpha.age~dnorm(0,0.0001)

alpha.sex~dnorm(0,0.0001)

τ_patient_~dgamma(1,1)

τ_0_~dgamma(1,1)

τ_age_~dgamma(1,1)

τ_sex_~dgamma(1,1)

Supplementary Figure 1


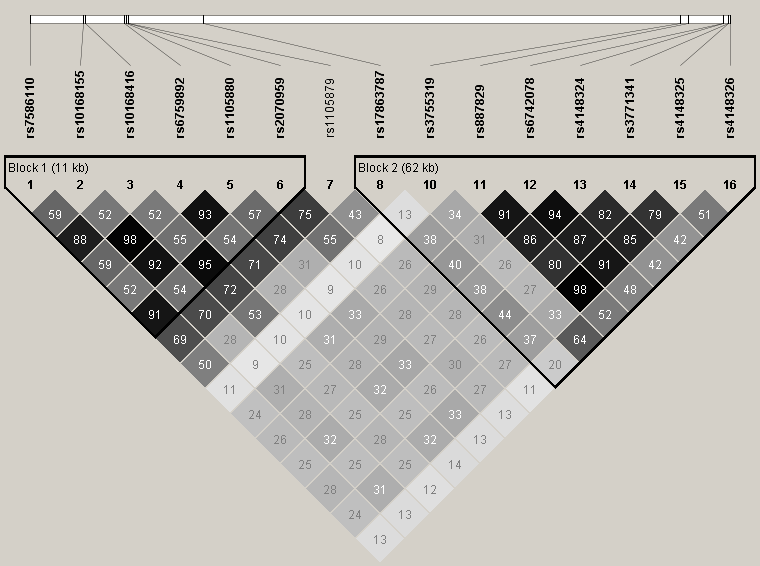


**Figure 1 LD Structure in CSSCD Cohort.** LD plots for regions in genes *UGT1A1, UGT1A3, UGT1A4, UGT1A5, UGT1A6, UGT1A7, UGT1A8, UGT1A9* and *UGT1A10* on chromosome 2 in the CSSCD subjects. The LD plot was generated using Haploview 4.2. Each diamond represents the r^2^ value between two SNPs. The LD color scheme is: white r^2^=0, 0<r^2^<1 grey (the darker the shade of grey, the higher the r^2^ value), black r^2^=1.

Adjusted Analysis

In order to determine if these associations were part of one or multiple signals we performed a SNP set association analysis adjusting for age, sex and our top SNP rs887829. The results can be seen in Supplementary Table 1. After adjusting for our top SNP, none of the SNPs show a significant association with serum bilirubin thus providing evidence that the associations shown in these 16 SNPs are part of one signal.

Supplementary Table 1

| SNP | Chr | BP | Coded Allele | NonCoded Allele | pvalue |
| --- | --- | --- | --- | --- | --- |
| rs7586110 | 2 | 234255266 | C | A | 0.5148 |
| rs10168155 | 2 | 234261575 | A | G | 0.9454 |
| rs10168416 | 2 | 234261826 | G | C | 0.3274 |
| rs6759892 | 2 | 234266408 | C | A | 0.9348 |
| rs1105880 | 2 | 234266704 | G | A | 0.8925 |
| rs2070959 | 2 | 234266930 | G | A | 0.2368 |
| rs1105879 | 2 | 234266941 | C | A | 0.05988 |
| rs17863787 | 2 | 234275833 | C | A | 0.2617 |
| rs3755319 | 2 | 234332321 | A | C | 0.1526 |
| rs6742078 | 2 | 234337378 | A | C | 0.8188 |
| rs4148324 | 2 | 234337461 | C | A | 0.1547 |
| rs3771341 | 2 | 234337978 | A | G | 0.9155 |
| rs4148325 | 2 | 234338048 | A | G | 0.5338 |
| rs4148326 | 2 | 234338201 | A | G | 0.6086 |
